# Supplementary material for: Quantifying early COVID-19 outbreak transmission in South Africa and exploring vaccine efficacy scenarios
Source: PLoS One. 2020 Jul 24;15(7):e0236003. doi: 10.1371/journal.pone.0236003 (PMC7380646; doi:10.1371/journal.pone.0236003)
Supplement: S1 Fig — The arrows connecting compartments denote COVID-19 infection at rate βS(t)I(t)/N, progression to infectiousness σE and recovery rate γI respectively. (DOCX) [file pone.0236003.s001.docx]

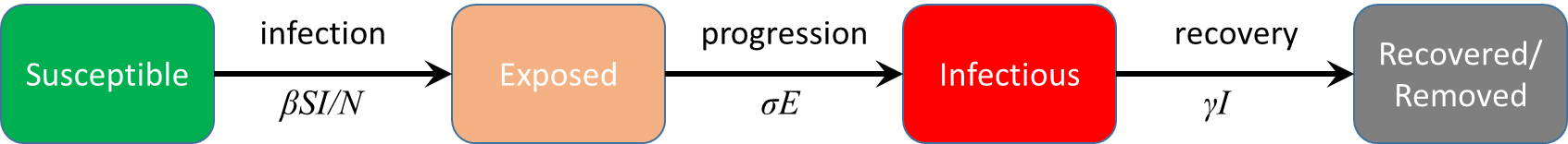


**S1 Fig:** Schematic COVID-19 model diagram outlining infection progression**.** The arrows connecting compartments denote COVID-19 infection at rate $\beta S\left( t \right)I\left( t \right)/N$, progression to infectiousness $\sigma E$ and recovery rate $\gamma I$ respectively.
