# Supplementary material for: Quantifying early COVID-19 outbreak transmission in South Africa and exploring vaccine efficacy scenarios
Source: PLoS One. 2020 Jul 24;15(7):e0236003. doi: 10.1371/journal.pone.0236003 (PMC7380646; doi:10.1371/journal.pone.0236003)
Supplement: S1 Table — This data of cases reported from the 5th March to 11th April 2020 data. (DOCX) [file pone.0236003.s002.docx]

**S1 Table:** **Cumulative COVID-19 cases for South Africa**. This data of cases reported from the 5^th^ March to 11^th^ April 2020 data.

| **Date** | **Cumulative COVID-19 cases** |
| --- | --- |
| 05 March 2020 | 1 |
| 06 March 2020 | 1 |
| 07 March 2020 | 2 |
| 08 March 2020 | 3 |
| 09 March 2020 | 6 |
| 10 March 2020 | 9 |
| 11 March 2020 | 13 |
| 12 March 2020 | 16 |
| 13 March 2020 | 24 |
| 14 March 2020 | 38 |
| 15 March 2020 | 51 |
| 16 March 2020 | 62 |
| 17 March 2020 | 85 |
| 18 March 2020 | 116 |
| 19 March 2020 | 150 |
| 20 March 2020 | 202 |
| 21 March 2020 | 240 |
| 22 March 2020 | 274 |
| 23 March 2020 | 402 |
| 24 March 2020 | 554 |
| 25 March 2020 | 709 |
| 26 March 2020 | 927 |
| 28 March 2020 | 1187 |
| 29 March 2020 | 1280 |
| 30 March 2020 | 1326 |
| 31 March 2020 | 1353 |
| 01 April 2020 | 1380 |
| 02 April 2020 | 1462 |
| 03 April 2020 | 1505 |
| 04 April 2020 | 1585 |
| 05 April 2020 | 1655 |
| 06 April 2020 | 1686 |
| 07 April 2020 | 1749 |
| 08 April 2020 | 1845 |
| 09 April 2020 | 1934 |
| 10 April 2020 | 2003 |
| 11 April 2020 | 2028 |
