# Supplementary material for: Quantifying early COVID-19 outbreak transmission in South Africa and exploring vaccine efficacy scenarios
Source: PLoS One. 2020 Jul 24;15(7):e0236003. doi: 10.1371/journal.pone.0236003 (PMC7380646; doi:10.1371/journal.pone.0236003)
Supplement: S2 Table — *We used population size for Gauteng, Western Cape, KwaZulu-Natal and Free State provinces in estimating national level estimates as these were the only provinces with COVID-19 cases in the early phases of the epidemic. Population estimates were based on the 2019 census [1]. (DOCX) [file pone.0236003.s003.docx]

**S2 Table**: **COVID-19 model parameters and values.** *We used population size for Gauteng, Western Cape, KwaZulu-Natal and Free State provinces in estimating national level estimates as these were the only provinces with COVID-19 cases in the early phases of the epidemic. Population estimates were based on the 2019 census [1].

| **Parameter description** | **Parameter symbol** | **Range/value** | **Reference** |
| --- | --- | --- | --- |
| ^*^Population Size | N | 36 196 938 | [1] |
| Effective contact rate | $\beta$ | 0-2 day^-1^ | assumed |
| Incubation period | $1/\sigma$ | 3-7 days | [2] |
| Infectious period | $1/\gamma$ | 2-5 days | [3, 4] |
| Percentage reduction in effective contacts | $\epsilon$ | 0-100% | assumed |

*S2 Table References*

| [1] | STATS SA, "Statistical release P0302, Mid-year population estimates, Retrieved March 30, 2020, from *http://www.statssa.gov.za/publications/P0302/P03022019.pdf*," 2019. |
| --- | --- |
| [2] | WORLDOMETER, "Corona Incubarion Period, Retrieved March 31, 2020, from *https://www.worldometers.info/coronavirus/*," 2020. |
| [3] | X. Li, X. Guan, P. Wu and et al., "Early transmission dynamics in Wuhan, China, of novel coronavirus–infected pneumonia, Retrieved April 11, 2020, from *https://www.nejm.org/doi/full/10.1056/NEJMoa2001316*," pp. p. published online Jan 29, 2020, 2020. |
| [4] | F. A. Hamzah Binti, C. Lau, H. Nazri and et al., "CoronaTracker, World-wide COVID-19 Outbreak data analysis and prediction," World Health Organisation, Retrieved April 10, 2020, from Bulletin of World Health Organisation, Geneva, 2020. |
